# Imageless navigation demonstrates limited anteversion agreement with postoperative EOS assessment in lateral decubitus total hip arthroplasty

**DOI:** 10.1007/s11701-026-03542-y

**Published:** 2026-06-01

**Authors:** Leandra Bauer, Philipp Knospe, Steffen Brodt, Georgi Wassilew, Georg Matziolis

**Affiliations:** 1 Experimental Orthopaedics, University Hospital Jena, Campus Eisenberg, Waldkliniken Eisenberg, Friedrich-Schiller-University Jena, Jena, Germany; 2 Orthopaedics, University Hospital Jena, Campus Eisenberg, Waldkliniken Eisenberg, Friedrich-Schiller-University Jena, Jena, Germany; 3 Department of Orthopaedic, University Hospital Greifswald, Greifswald, Germany

**Keywords:** Total hip arthroplasty, Imageless navigation, Lateral decubitus position, Acetabular component positioning

## Abstract

**Introduction:**

Imageless navigation is widely used in total hip arthroplasty (THA), yet evidence for procedures in the lateral decubitus position remains limited because pelvic orientation and registration differ from the supine position. This study evaluated the accuracy of imageless navigation for acetabular component positioning in lateral-position primary THA, using postoperative EOS-based 3D assessment as a postoperative reference method for agreement analysis.

**Methods:**

The study comprised in-vitro pretests and an in-vivo cohort. In vitro, a pelvic model was systematically rotated along all axes to assess effects on navigated cup inclination and anteversion. In vivo, 70 patients undergoing primary THA in lateral decubitus were included. Intraoperative imageless navigation values were compared with postoperative EOS-3D-measurements.

**Results:**

In vitro, z-axis (tilt) variations substantially altered both parameters. In vivo, inclination showed a small but statistically significant inter-method difference (mean 1.4°, p = 0.036, Cohen’s d = 0.26), whereas anteversion demonstrated a larger systematic underestimation by imageless navigation (mean −7.5°, p < 0.001, Cohen’s d = −0.78) with poor inter-method agreement (ICC = 0.168).

**Conclusion:**

Imageless navigation demonstrated acceptable inclination agreement with postoperative EOS assessment, whereas anteversion showed a larger systematic deviation and poor inter-method agreement; sagittal pelvic tilt and positional frame-of-reference differences appear to be major contributing factors.

**Clinical trial registration:**

The study was registered in the German Clinical Trials Register with the registration number DRKS00026749.

## Introduction

Total hip arthroplasty is among the most frequently performed and most successful procedures in orthopaedic surgery, with steadily increasing implantation numbers worldwide. Despite its overall success, the accurate positioning of the acetabular component remains a critical determinant of surgical outcomes, directly influencing postoperative stability, range of motion, implant longevity, and patient satisfaction [3, 5, 17, 19, 20]. Pelvic orientation and precise acetabular cup placement are paramount, as even small angular deviations can lead to significant clinical consequences including dislocation, impingement, accelerated polyethylene wear, and limited functional outcomes [5, 8, 17].

The concept of a “safe zone” for acetabular component positioning, originally proposed by Lewinnek et al., has traditionally guided surgeons toward target inclination angles of 40°±10° and anteversion angles of 15°±10° relative to the anterior pelvic plane [10]. Recent evidence has evolved this understanding toward patient-specific or functional safe zones that account for individual hip-spine relationships and pelvic mobility, recognizing that optimal component orientation may vary based on patient anatomy and functional demands [4].

Conventional THA techniques rely primarily on mechanical alignment guides, anatomical landmarks, and surgeon experience to determine acetabular component orientation. While these methods are cost-effective and straightforward to implement, they suffer from substantial intercase variability and produce notable outliers that fall outside acceptable positioning parameters [2].

Imageless navigation systems employ various registration strategies and sensor technologies to establish spatial orientation of the pelvis and provide angular guidance for cup placement. The fundamental principle involves creating a coordinate system based on either anatomical landmarks (anterior superior iliac spines and pubic symphysis defining the anterior pelvic plane) or functional references (such as table tilt or gravity-based axes), then tracking the position of surgical instruments relative to this reference frame in real time [14, 15].

Multiple studies have demonstrated measurable improvements in acetabular component positioning accuracy and consistency with imageless navigation compared to conventional techniques. Cadaver and clinical comparative studies consistently show reduced variability and tighter positioning ranges with imageless navigation assistance [7, 11, 16].

However, despite this progress, current applications of imageless navigation in THA have largely focused on procedures performed in the supine position. This is a noteworthy observation, given that THA can be performed with the patient in a variety of positions, depending on the surgical approach. Although supine positioning is customary for anterior or anterolateral approaches, the lateral decubitus position remains a widely employed technique for posterior or lateral approaches. Consequently, the anatomical reference frames and mechanical constraints influencing pelvic tilt, femoral mobility and intraoperative registration differ substantially between positions. In addition, it is known that the pelvic position can vary greatly during surgical positioning [6]. The reliability of imageless navigation systems under these varying conditions is evidenced by limited research, despite the continued application of the lateral decubitus position in clinical practice.

The present study aims to address this gap by examining the reliability and accuracy of imageless navigation when total hip arthroplasty is performed with the patient in the lateral decubitus position. The objective of this study is to evaluate the performance of imageless navigation under specific conditions in order to ascertain whether the advantages reported for navigated procedures in the supine position can be replicated across alternative patient positions that are integral to established surgical approaches in hip arthroplasty.

## Materials and methods

The study was approved by the local ethics committee (IRB no. 2021-2165-BO), and all participants signed an informed consent. All methods were performed in accordance with the relevant guidelines and regulations. The study was registered in the German Clinical Trials Register with the registration number DRKS00026749.

### In-vitro pre-tests for sensitivity analysis

To evaluate the robustness of the imageless navigation system and assess the influence of pelvic malalignment on measurement accuracy, a series of controlled in vitro tests were conducted prior to clinical application. These experiments aimed to simulate variations in pelvic tilt and rotation by systematically altering the orientation of the pelvic reference frame in all three anatomical planes.

A standardized biomechanical pelvic model (Sawbone, Pacific Research Laboratories, Vashon, WA, USA) was rigidly fixed in a dedicated holding fixture to ensure reproducible positioning. Pelvic orientation was systematically varied along each anatomical axis using defined angular increments. Reference angles were independently verified using a three-dimensional optical measurement system (GOM Aramis, Carl Zeiss GOM Metrology, Braunschweig, Germany), which served as the external reference for angular calibration. The anterior pelvic plane, defined by the anterior superior iliac spines and the pubic symphysis, was used as the pelvic reference frame throughout all in-vitro measurements.

Using that standardized setup, the pelvic model was incrementally tilted along the x-axis (axial rotation), y-axis (lateral tilt), and z-axis (sagittal tilt) to replicate potential intraoperative deviations from the neutral pelvic position. For each variation, acetabular component inclination and anteversion were recorded using the imageless navigation system (NaviSwiss AG, Brugg, Switzerland). This approach allowed for a systematic analysis of how positional deviations affect the reliability of imageless navigation measurements.

### In-vivo setup and data acquisition

#### Patients and surgery

Patients scheduled for primary THA due to symptomatic end stage hip osteoarthritis were considered for inclusion in this study. Only individuals with primary osteoarthritis of the hip were eligible. Patients were excluded if they exhibited secondary osteoarthritis, hip fractures, bone or soft tissue tumors involving the hip region, or any condition necessitating revision arthroplasty. Additional exclusion criteria were age below eighteen years and pregnancy.

All patients received one of two cementless femoral stems commonly used in contemporary THA: either the Fitmore B stem (Zimmer Biomet, Warsaw, IN, USA) or the Optimys stem (Enovis, Wilmington, DE, USA). A cementless acetabular cup (Allofit, Zimmer Biomet, Warsaw, IN, USA) with a ceramic femoral head and highly cross-linked polyethylene liner was used in all cases. All procedures were performed by one of two experienced senior orthopaedic surgeons.

**Fig. 1:**
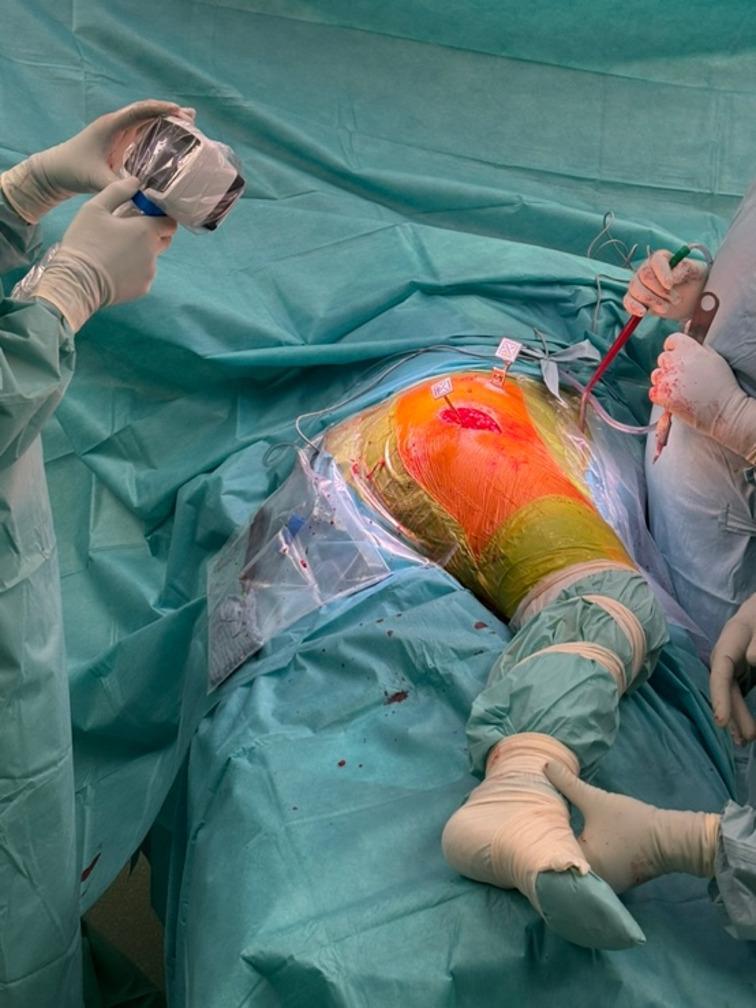
Intraoperative of the NaviSwiss imageless navigation system during primary total hip arthroplasty in the lateral decubitus position, with optical trackers attached to the pelvis and femur for real-time assessment of acetabular component orientation

Surgery was performed with all patients placed in the lateral position. An imageless hip navigation system (NaviSwiss AG, Brugg, Switzerland) was used for every procedure. After routine surgical preparation and draping, optical markers were attached to the pelvis and the femur according to the manufacturer’s instructions, followed by calibration of the imageless navigation system (Fig. 1). All procedures were performed using a minimally invasive posterior approach, with the imageless navigation workflow standardized across both surgeons according to the manufacturer’s protocol. Acetabular component inclination and anteversion were recorded intraoperatively using the imageless navigation platform. Subsequently, a cementless acetabular cup and the selected cementless femoral stem were implanted in standard fashion.

#### Post-OP radiological setup

On the day of discharge, all patients underwent standardized full weight bearing imaging using the EOS system (EOS^®^ imaging, Paris, France). The acquisition was performed in an upright standing position to capture the alignment of the pelvis and lower extremities under physiological load. The resulting biplanar radiographs were subsequently processed to generate three-dimensional reconstructions of the relevant bony structures, including the pelvis and femur using the EOS Software (EOS^®^ imaging, Paris, France).

These 3D models were then analyzed to determine the orientation of the acetabular component. Anteversion and inclination were measured directly within the reconstructed 3D object, allowing for a precise assessment of implant position in relation to the patient’s functional posture.

#### Data analysis and statistics

For each patient, measurements of acetabular component orientation obtained intraoperatively from the imageless navigation system and postoperatively from EOS imaging were included in the analysis. To assess potential differences between the two methods, paired t-tests were performed for both anteversion and inclination using SPSS (Version 30, IBM Corp., Armonk, NY, USA), with the level of statistical significance set at *p* = 0.05. Normal distribution of the paired differences was assessed using the Kolmogorov–Smirnov test with Lilliefors correction. The differences for both inclination and anteversion showed no significant deviation from normal distribution using the Kolmogorov–Smirnov test with Lilliefors correction (both *p* ≥ 0.200), supporting the use of paired t-tests.

Effect sizes for paired comparisons were calculated using Cohen’s d with 95% confidence intervals to support interpretation of the magnitude of inter-method differences. Inter-method agreement between imageless navigation and EOS assessment was quantified using intraclass correlation coefficients (ICC) with 95% confidence intervals. ICCs were calculated using a two-way mixed-effects model for absolute agreement.

Agreement between imageless navigation and EOS measurements was further evaluated using Bland–Altman plots, generated in MATLAB (MathWorks, Natick, MA, USA), to identify systematic biases. Additionally, linear regression analyses were conducted to examine correlations between navigated and radiologically assessed values.

## Results

### In-vitro pre-tests

The experimental simulations revealed that variations in pelvic orientation around the x-axis (rotation) and y-axis (lateral tilt), corresponding to pelvic rotation and tilt respectively, had no measurable effect on the acetabular component orientation as recorded by the imageless navigation system. Both inclination and anteversion values remained stable across these conditions.

In contrast, alterations in the pelvic position around the z-axis, representing axial pelvic tilt (anterior or posterior), had a substantial impact on the measured acetabular orientation. With increasing posterior tilt (negative z-axis rotation), both inclination and anteversion values increased markedly. At a neutral position (0°), inclination was measured at 45° and anteversion at 26°. A posterior tilt of − 20° resulted in an inclination of 56° and anteversion of 39°, whereas an anterior tilt of + 10° reduced inclination to 42° and anteversion to 17° (see Table 1).

**Table 1:** Results of variation of z-axis (tilt) for inclination and anteversion in imageless navigation setup

z-axis (Tilt)	**Inclination [°]**	**Anteversion [°]**
-20	56	39
-15	53	36
-10	50	32
0	45	26
10	42	17

### In-vivo patient data

A total of 70 THA patients were included in the study, comprising 30 left and 40 right hips. Patients received either the Optimys or Fitmore cementless femoral stem. The mean femoral offset was 46.6 ± 13.0 mm (range: 27.0–111 mm), and mean stem antetorsion was 8.2 ± 10.8° (range: −24.7° to 28.7°); cup sizes ranged from 48 to 62 mm (mean 54.5 mm).

Table 2 summarizes the comparison of imageless navigation-based measurements and postoperative EOS-based assessments. Mean inclination measured intraoperatively with the imageless navigation system was 42.0 ± 4.7° (range: 32.0–52.0°), compared to 40.6 ± 6.3° (range: 24.0–53.8°) postoperatively on EOS imaging. The mean paired difference was 1.4° ± 5.4° with a 95% confidence interval of 0.1° to 2.7°, indicating a small but statistically significant difference between methods (*p* = 0.036). The corresponding effect size was small (Cohen’s d = 0.26; 95% CI, 0.02 to 0.49). The mean anteversion was 21.6 ± 4.8° (range: 11.0–34.0°) with imageless navigation and 29.0 ± 9.9° (range: 7.2–48.0°) with EOS imaging. The mean paired difference was − 7.5 ± 9.6° with a 95% confidence interval of −9.7 to −5.2°, indicating a larger systematic underestimation of anteversion by imageless navigation (*p* < 0.001). The corresponding effect size was moderate to large (Cohen’s d = − 0.78; 95% CI, − 1.05 to − 0.51).

**Table 2:** Comparison of acetabular cup inclination and anteversion measured intraoperatively with imageless navigation and postoperatively using EOS-based 3D assessment

	Imageless navigation [Min – Max]	EOS assessment [Min – Max]	Mean difference Nav – EOS (95% CI)	Cohen’s d (95% CI)	*p*-Value
**Inclination [°]**	42.0 ± 4.7 [32.0–52.0]	40.6 ± 6.3 [24.0–53.8]	1.4 ± 5.4 (0.1 to 2.7)	0.26 (0.02 to 0.49)	**0.036**
**Anteversion [°]**	21.6 ± 4.8 [11.0–34.0]	29.0 ± 9.9 [7.2–48.0]	−7.5 ± 9.5 (−9.7 to −5.2)	−0.78 (−1.05 to −0.51)	**< 0.001**

Values are presented as mean ± SD. Mean differences are reported as navigation minus EOS assessment with corresponding 95% confidence intervals and p-values from paired t-tests, bold indicates significant differences (p < 0.05)

Based on ICC analysis, inclination demonstrated moderate inter-method agreement, whereas anteversion demonstrated poor agreement. The ICC for inclination was 0.485 (95% CI, 0.286 to 0.644), and the ICC for anteversion was 0.168 (95% CI, − 0.046 to 0.376).

**Fig. 2:**
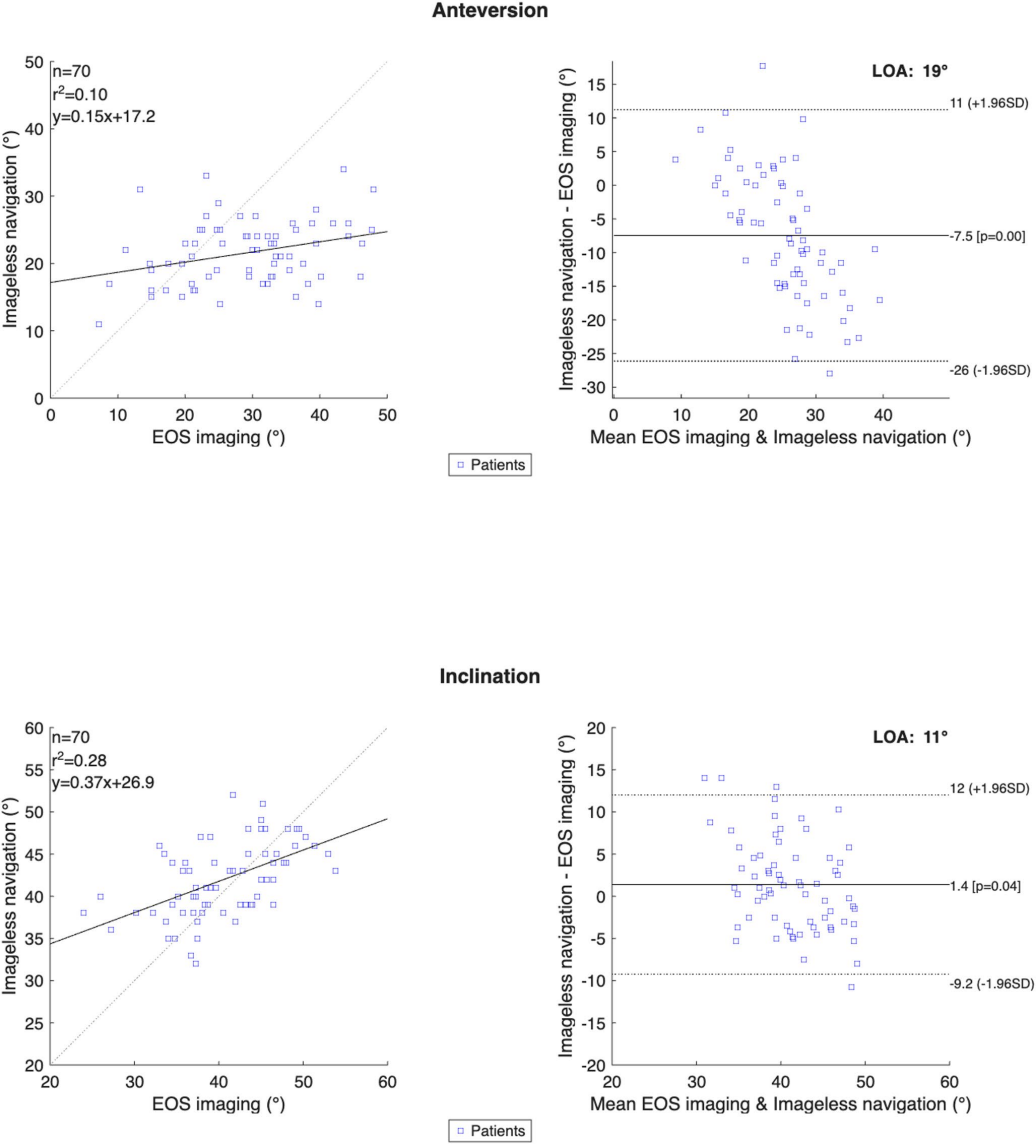
Linear regression and Bland–Altman plots comparing acetabular cup anteversion and inclination measured with intraoperative imageless navigation and postoperative EOS-based 3D assessment. Regression plots show EOS values on the x-axis and navigation values on the y-axis; the dashed line indicates the line of identity. Bland–Altman plots show the mean bias and 95% limits of agreement. Mean bias was − 7.5° for anteversion and 1.4° for inclination, with limits of agreement of − 26.1° to 11.2° and − 9.2° to 12.0°, respectively

Linear regression analysis (see Fig. 2) revealed a weak correlation between imageless navigation and EOS values for anteversion (R² = 0.10), while inclination demonstrated a moderate correlation (R² = 0.28). The regression lines deviated from the line of identity in both cases, indicating discrepancies between intraoperative and postoperative measurements.

Bland–Altman analysis confirmed a systematic underestimation of anteversion by the imageless navigation system, with a mean bias of − 7.5° with 95% limits of agreement (LoA) ranging from − 26° to 11°. For inclination, the mean difference was 1.4°, with 95% LoA between − 9.2° and 12°.

These findings indicate that while inclination measurements showed acceptable agreement between imageless navigation and EOS, anteversion values displayed greater variability and a consistent systematic deviation.

## Discussion

The main findings of the study indicate that imageless navigation in primary lateral decubitus THA showed a small but statistically significant difference for inclination and a substantially larger systematic underestimation of anteversion when compared with postoperative EOS-based 3D assessment. While in-vitro pretests demonstrated robustness of the system against pelvic rotation and lateral tilt, they also revealed a pronounced sensitivity to sagittal pelvic tilt (z-axis), with substantial shifts in both measured inclination and anteversion. This experimental observation is consistent with the in-vivo data, where inclination showed a small but statistically significant difference between methods, whereas anteversion was significantly lower intraoperatively and showed weak correlation with postoperative values.

The statistically significant difference in inclination should be interpreted in light of its small magnitude. The mean difference between imageless navigation and EOS assessment was only 1.4°, with a 95% confidence interval ranging from 0.1° to 2.7°. Such a small angular difference is unlikely to be clinically meaningful when considered in isolation, particularly compared with commonly accepted target ranges for acetabular inclination. In contrast, the mean anteversion difference of approximately 7.5° was larger, showed wider variability, and is therefore more relevant for clinical interpretation.

Together, these results suggest that the imageless navigation system showed closer agreement for inclination than for anteversion, but that anteversion assessment is more strongly influenced by pelvic tilt-related reference-frame effects and by differences between intraoperative lateral positioning and postoperative functional standing assessment. Therefore, intraoperative anteversion values should be interpreted cautiously in clinical practice. This interpretation is further supported by ICC analysis, which demonstrated moderate agreement for inclination but poor agreement for anteversion. Thus, although inclination differed statistically between methods, the magnitude of the mean difference was small and inter-method agreement was clearly better than for anteversion.

A central aspect of our study is that all procedures were performed in the lateral decubitus position, which directly addresses a gap in the imageless navigation literature. Most earlier evidence has evaluated imageless navigation without consistently stratifying by patient position, even though pelvic reference behavior differs fundamentally between supine and lateral setups. In our cohort, inclination showed acceptable agreement between imageless navigation and postoperative EOS-based 3D assessment, whereas anteversion was systematically underestimated. This position-specific pattern is clinically relevant because it suggests that accuracy claims derived from mixed-position cohorts may not be directly transferable to lateral decubitus THA.

This interpretation is supported by how prior studies were designed. The meta-analyses by Liu et al. [12] and Migliorini et al. [13] report no clear superiority of imageless navigation in mean cup angles, but both aggregate heterogeneous surgical settings and do not provide a robust position-stratified analysis. Migliorini et al. explicitly acknowledge heterogeneity in surgical approach/exposure [13].

In contrast, Nogler et al. [14] demonstrated reduced variability with imageless navigation in a cadaver model performed in the supine position, which confirms technical consistency but limits direct extrapolation to lateral decubitus surgery.

Position-specific contemporary evidence is provided by Okamoto et al. [15], who studied lateral decubitus THA and similarly found that anteversion remained the more error-prone parameter, with no accuracy advantage of accelerometer-based portable navigation over a pelvic alignment guide.

Our in-vitro findings provide a mechanistic explanation for these clinical observations: while rotation and lateral tilt had little effect, sagittal pelvic tilt markedly altered measured cup orientation, particularly anteversion. In lateral decubitus THA, where pelvic fixation and intraoperative manipulation can induce tilt changes, this sensitivity is likely amplified. Therefore, our data suggest that the main unanswered question is not whether imageless navigation is generally useful, but whether its reference strategy is sufficiently robust for lateral-position pelvic dynamics. From this perspective, our study extends prior literature by demonstrating that position is not a minor methodological detail, but a determinant of measurement validity, especially for anteversion when evaluated against postoperative functional imaging.

A key methodological aspect of the present study is the comparison of intraoperative measurements acquired in lateral decubitus position with postoperative EOS measurements obtained in upright standing posture. This represents an inherent coordinate-system mismatch. Pelvic orientation may differ substantially between surgically fixed lateral positioning and functional standing posture, particularly in the sagittal plane. Consequently, the observed anteversion difference may reflect a combination of navigation-related measurement behavior, sagittal pelvic tilt changes, and physiological postural differences rather than isolated navigation inaccuracy. Recent studies and reviews report that robotic-arm-assisted THA can improve radiographic accuracy of acetabular cup placement compared with conventional techniques, although the translation into superior patient-reported outcomes and cost-effectiveness remains less certain. A 2025 study comparing computer-assisted systems reported the highest cup placement accuracy with robotic assistance compared with CT-based and portable navigation systems [9], and a 2024 prospective cohort study evaluated robotic-arm-assisted THA with a focus on cup-positioning precision [18]. Recent meta-analytic evidence also suggests improved radiological cup placement with robotic THA, while clinical superiority remains less consistently demonstrated [1].

Several limitations should be considered when interpreting our findings. First, although EOS-based 3D analysis was used as the postoperative reference method for agreement analysis, EOS itself is not error-free. Although EOS-based 3D assessment provides clinically relevant postoperative evaluation under weight-bearing conditions, it should not be interpreted as a definitive gold standard. Measurement uncertainty may arise from image acquisition, landmark identification, reconstruction algorithms, and observer-dependent segmentation, particularly in the presence of metallic implants. Second, potential 3D measurement error in EOS is not uniform across parameters: based on the known geometric sensitivity of cup orientation analysis, error is typically lower for inclination and can be higher for anteversion, where small changes in pelvic orientation or landmark definition may translate into larger angular deviations. This is important for our interpretation, because part of the observed imageless navigation–EOS difference in anteversion may reflect compounded error from both methods rather than imageless navigation bias alone. Third, our comparison links an intraoperative, position-dependent imageless navigation coordinate system (lateral decubitus) to a postoperative functional standing EOS assessment; this is clinically meaningful but introduces a systematic frame-of-reference mismatch that cannot be fully eliminated. Finally, the study was conducted at a single center with one imageless navigation platform and specific surgical workflows, which may limit generalizability to other institutions, implant systems, and registration techniques.

## Conclusion

In lateral decubitus THA, imageless navigation showed a statistically significant but small mean difference for cup inclination compared with postoperative EOS-based 3D assessment. In contrast, anteversion demonstrated a larger systematic deviation and wider variability. These findings suggest that inclination measurements may remain clinically interpretable, whereas anteversion values require particular caution when intraoperative lateral-position navigation is compared with postoperative functional standing assessment.

## Data Availability

No datasets were generated or analysed during the current study.
